# Resemblance in accelerometer-assessed physical activity in families with children: the Lolland-Falster Health Study

**DOI:** 10.1186/s12966-020-01067-7

**Published:** 2020-12-04

**Authors:** Therese Lockenwitz Petersen, Jan Christian Brønd, Peter Lund Kristensen, Eivind Aadland, Anders Grøntved, Randi Jepsen

**Affiliations:** 1 Department of Sports Science and Clinical Biomechanics, Research Unit for Exercise Epidemiology, Centre of Research in Childhood Health, University of Southern Denmark, Campusvej 55, DK-5230 Odense M, Denmark; 2 Lolland-Falster Health Study, Centre for Epidemiological Research, Nykøbing F. Hospital, Fjordvej 15, 4800 Nykøbing F., Denmark; 3 University College Absalon, Bispegade 5, 4800 Nykøbing F., Region Zealand Denmark; 4 Faculty of Education, Arts and Sports, Department of Sport, Food and Natural Sciences, Western Norway University of Applied Sciences, Røyrgata 4, 6856 Sogndal, Norway

**Keywords:** Physical activity, Family, Children, Parents, Parent-child dyads, Accelerometer, Clustering, Lolland-Falster Health Study, LOFUS, Denmark

## Abstract

**Background:**

Evidence of intra-family resemblance in physical activity (PA) is lacking. The association between parent and child PA appears weak, the influence of age and gender on this association is uncertain, and no studies have investigated the degree of resemblance in family members’ PA behaviours such as walking, sitting/lying, and biking. Thus, the aims of the study were to examine the degree of resemblance in PA within families, specifically between parents and children, and to explore the size of resemblance across age of children, gender of parents and children, and intensity and type of PA.

**Method:**

The study is a cross-sectional analysis of a subsample (902 parents and 935 children nested within 605 families) of the Danish population study Lolland-Falster Health Study. PA was measured using a dual-accelerometer system (Axivity AX3) with subsequent processing of time spent in light PA (LPA), moderate-to-vigorous PA (MVPA), and vigorous PA and classification of PA behaviour types. Families with at least one son/daughter aged 0–22 years and one parent providing minimum 4 days of valid accelerometer data were included in the analysis. A linear mixed model regression analysis was used to determine the intraclass correlation coefficient (ICC) of clustering among family members for PA intensities and PA behaviours, adjusted for sex, age, parental education, and the interaction between sex and age.

**Results:**

In the analysis of within-family variation in PA, the ICCs across PA intensities and PA behaviours ranged from 0.06 to 0.34. We found stronger clustering in family members’ PA for LPA and behaviours requiring low energy expenditure (LPA: ICC 0.22 (95% confidence interval (CI) 0.17; 0.28), sitting/lying: ICC 0.34 (95% CI 0.28; 0.40)), and walking: ICC 0.24 (95% CI 0.19; 0.30) than for higher intensities (e.g. MVPA: ICC 0.07 (95% CI 0.03; 0.14)). The ICC for biking was 0.23 (95% CI 0.18; 0.29). Analyses on parent-child dyads gave similar results. No interaction effects for gender and age (except for biking) were found.

**Conclusion:**

Parents and children’s time spent in PA behaviours requiring low energy expenditure had moderate resemblance within families, whereas engagement in PA with higher intensities showed small or close-to-zero resemblance.

## Introduction

Physical activity (PA) is a complex behaviour influenced by multiple individual, inter-relational, social, environmental, and political factors [1]. On the inter-relational level, family is an entity and an arena for connectedness and interactions among parents and children [2]. Interactions within a family are complex and multidirectional, and family members may influence each other in health-related behaviours, through for example norms, routines, negotiations, prioritisation, resistance, and cooperation [2, 3]. Thus, the family is considered to play a role for the PA of the family as a whole and for the PA of each individual family member [1, 4].

The physical and mental health benefits of PA across the life course are well established [5, 6], which in the family setting means that PA is important for both parents and children. The PA of family members may include non-structured (e.g. childcare, housework, playing, and watching TV) and structured activities (e.g. sport, exercise, and transportation). The diverse activities within the family setting require different PA behaviours such as sitting, standing, walking, and running and thus, include various PA intensities [7]. Parents and children carry out some of these activities together, whereas others are performed individually in shared or non-shared settings [3]. Especially for boys, intra-family PA patterns may have specific implications for future activity levels, because their childhood PA behaviours seem to track moderately into adulthood. For girls, this tracking tends to be lower. Growing up, the most crucial age for adulthood PA levels seems to be adolescence [8]. In the family PA context, much previous research have focused on parents being important socialisation agents influencing children’s PA behaviour through e.g. parenting style, shared environment, and co-participation [9, 10]. Genetics seem to have relatively low influence on the variability of intra-family PA levels compared to environmental factors [11].

In a recent systematic review on associations between parents’ device-measured or self-reported PA and children’s device-measured PA, the authors found a weak, positive relationship between parent and child PA across all studies (average correlation of 0.13) [12]. However, the internal validity of the included studies was generally low (high risk of bias), which may blur the size of estimated average magnitude of correlation between parent and child PA. For example, studies using self-reported data on parental PA showed a weaker relationship between parent and child PA than studies using device-based measures (average magnitude of correlation 0.04 versus 0.16, respectively). Moreover, while the included studies examined associations between PA intensities (especially moderate-to-vigorous PA (MVPA)) of parents and children, the authors of the review did not identify studies that examined associations for time spent in specific types of PA behaviours such as walking, sitting, lying, and biking. Further, there is a lack of knowledge about the impact of child age [13] and inconsistent findings about the potential influence of gender [14], which calls for further examination. Thus, more high-quality research is needed to improve our understanding of the co-occurrence (i.e. clustering) of time spent in specific PA intensities and PA behaviours among family members, specifically among parents and children. In a public health perspective, more knowledge about the family setting as one of many contributors to PA as a complex behaviour may inform health promotion initiatives.

Therefore, the aims of this study were 1) to examine the degree of resemblance in PA within families with children and between parents and children, and 2) to explore the degree of resemblance across age of children, the gender of parents and children, and the intensity and type of PA.

## Method

### Study design and participants

We used data from a subsample of households in the Lolland-Falster Health Study (LOFUS) [15]. In brief, LOFUS is a Danish household-based population study that enrolled 19,000 participants aged 0–96 years between 8 February 2016 and 13 February 2020. Randomly selected individuals aged ≥18 years living on one of the two Danish islands Lolland and Falster and, if any, their household members were invited to participate and contribute with data for many research purposes. Participation was voluntary for each household member. The data collection encompassed questionnaires, a site visit including a series of physical examinations, and collection of biological samples [15]. At the end of the site visit, a subsample of LOFUS participants was asked to wear accelerometers. Between 1 February 2017 and 30 November 2018, the inclusion criteria was that at least one child and one adult from a given household should agree to accelerometer assessment. From 1 December 2018 to 13 February 2020, all LOFUS participants were eligible for inclusion. Subjects who could not walk were excluded. The present study included families (with children up to the age of 22 years), who participated in LOFUS between 1 February 2017 and 2 October 2019. We operationalised *family* as at least one parent and at least one child from the same household. *Parent* refers to a primary caregiver, which could be a biological parent, a stepparent, a foster parent, or any other legal guardian [16].

### Socio-demographic information

Socio-demographic information about the parents was obtained by self-reported questionnaires [17]. Data on civil status was dichotomised as 1) married/cohabiting and 2) divorced/separated/single/widow(er). Educational level was divided into three categories: 1) medium (3–4 years) or longer higher education (≥5 years), 2) short higher education (2–3 years) or vocational education, and 3) one or multiple shorter courses or no formal education. In families including two participating parents, the highest reported educational level was used as parental education in the analyses. Sixteen response options on occupational status were categorised as being 1) employed (e.g. employees, employers, or self-employed), 2) student (e.g. in high school, college, or vocational training), and 3) out of work (e.g. on social benefits or unemployed).

### Anthropometry

Anthropometric measures were taken at the site visit. Height and weight were obtained using standardized anthropometric procedures [15], and body mass index was calculated.

### Measurement of physical activity

Two Axivity AX3 accelerometers [18] were used to measure PA in parents and children. The accelerometers were attached to the skin using adhesive plaster to enable full 24-h recording. One accelerometer was worn on the right side of the lower back above the pelvic ridge, and the other on the front of the right thigh in the midst between the knee and the hip. The participants were instructed to wear the accelerometers for seven consecutive days, including during sleep and water activities and to reapply the accelerometers if they fell off.

### Data reduction of raw accelerometer data

Valid wear periods were identified by evaluating acceleration and temperature. The raw acceleration was band-pass filtered (0.1–4 Hz) and temperature low pass filtered (0.05 Hz) using a fourth order Butterworth filter (zero delay). A non-moving temperature (NMT) threshold was individually determined from the temperature recorded during movement (the lower limit of the 95% confidence interval (CI)). Periods of no movement (consecutive acceleration below 20 mg) longer than 120 min were always identified as non-wear, and shorter periods from 45 to 120 min were identified as non-wear if the average temperature was below the estimated NMT threshold. Periods of 10 to 45 min with no movement were only identified as non-wear if the average temperature was below the NMT threshold, and if the end of the period was within the expected awake time (6:00 a.m. to 10:00 p.m.). Periods with active movement were identified as device transport (device moving but not worn by the subject) if the average temperature was below the NMT threshold [19]. PA intensity was determined by generating ActiGraph counts using 10 s-epochs from the raw acceleration measured at the back [20]. The PA intensity estimated using counts with intermittent activities is known to be underestimated [21, 22] and in order to account for this measurement error, we identified high intensity bouts using vector magnitude acceleration separated with no movements of less than 10 s and used interpolation to account for the elevated post oxygen consumption between bouts [23]. The known measurement error with intermittent activities [21, 22] was reduced by interpolating the non-moving activity between closely related (< 10 s distance) bouts of high intensity (vigorous) activity bouts (Brønd JC, Andersen LB, Grøntved A, Pedersen HA, Arvidsson D: Accurate assessment of intermittent activity with accelerometry, submitted). This method is similar to the two-regression method originally proposed by Crouter et al. [24], however avoiding the use of multiple regressions equations and improving the resolution by correcting the measurement error in the second-by-second data [24–26]. Criteria for a valid day was at least 8 h of wear time [27–29]. The awake time analysis was restricted to 6:00 a.m. and 11:59 p.m. (both weekdays and weekend days), and only subjects providing at least 4 days were included. Furthermore, only households that provided valid data from at least one parent (≥23 years) and one child (0–22 years) were included in the final analyses. Estimating subjects' time spent in the commonly used intensity intervals light (LPA), moderate (MPA), vigorous (VPA), and moderate-to-vigorous intensity (MVPA) was based on application of count thresholds. Activity counts specific intensity thresholds were established using an internally conducted validation experiment. The experiment included 133 subjects in the age range of 5 to 50 years. The subjects were divided into a pre-school group (5–6 years, *N* = 29), a child group (9–11 years, *N* = 35), an adolescent group (14–16 years, *N* = 31), and an adult group (> 18 years, *N* = 38). Identifying age-independent MPA and VPA intensity-specific counts thresholds is challenging, and adjusting for basal metabolic rate to account for body weight, height, and maturation is not an accurate solution [30]. The metabolic and mechanical cost of walking at self-selected speed is similar across a large age range [31] and performed at an intensity corresponding to 30–35% of individuals’ VO_2max_. Moreover, the metabolic cost of running is performed at an intensity > 60% of VO_2max_, suggesting that running at any speed requires a vigorous effort. This suggests that a moderate intensity cut-point can be defined as the average counts for walking at self-selected speed irrespective of age, whereas the vigorous cut-point as the counts threshold at which most subjects are considered running (the lower limit of the 95% CI). The moderate cut-points for the four groups were 1680, 3075, 3522, and 3522, whereas the vigorous cut-points were 3368, 5543, 5755, and 6016 [22]. MVPA was calculated as the sum of MPA and VPA activity. The LPA cut-point was set to 100 counts for all age groups. Estimating the time spent lying, sitting, standing, walking, biking, and running for each subject was determined using the method described by Skotte et al. [32]. This method uses a simple decision tree in combination with six different signal features generated from the thigh and the back raw acceleration data to identify PA behaviours. In the study by Skotte et al., a very high sensitivity and specificity was demonstrated with the identification of several PA behaviours [32]. The method has been evaluated with children aged five to 16 years, and the results from the study demonstrate similar sensitivity and specificity as with adults [33].

### Ethics

Region Zealand’s Ethical Committee on Health Research (SJ-421) and the Danish Data Protection Agency (REG-24-2015 and REG-147-2016) approved the study. LOFUS is registered in Clinical Trials (NCT02482896). Written informed consent was obtained at the site visit. The holders of custody signed the consent form for participants aged 0–14 years [15].

### Statistics

The statistical analyses were conducted using Stata version 16.0 (StataCorp, College Station, Texas, USA).

Subject characteristics are presented as percentages for categorical variables and means ± standard deviations (SD) for continuous variables. Initially, we produced scatter plots of parent and child PA to graphically examine any possible departures from linearity. With no indications of non-linear relationships, mixed linear regression analysis with maximum likelihood estimation was used to estimate the clustering of PA within the total family and within parent-child dyads (random effect), adjusted for sex, age, parental education, and the interaction between sex and age (fixed effects). We also statistically tested the null hypothesis that the variance explained by the random effect was zero using a restricted likelihood ratio test comparing the random effect model with ordinary regression model. Based on the variance components of the estimated random effects (family or parent-child dyads), we calculated the intraclass correlation coefficient (ICC) with 95% CI as the ratio of the within-family (or within parent-child dyads) to total variance of PA. Thus, the ICC is a measure of resemblance within a cluster (e.g. the total family or parent-child dyads); an ICC closer to 1.00 indicates higher resemblance [34]. We refrained from calculating CI for low ICC values, due to close-to-zero denominator problem in the calculation of 95% CI with the delta method. A *p* value of ≤0.05 was considered statistically significant.

We estimated the degree of resemblance in PA among all family members (i.e. the total family) and the following parent-child dyads 1) one parent and one child, 2) one parent and one child aged 0–6 years, 3) one parent and one child aged 7–11 years, 4) one parent and one child aged 12–22 years, 5) one father and one daughter, 6) one father and one son, 7) one mother and one daughter, and 8) one mother and one son. If more than one parent and/or one child were eligible for a dyad, we made a random selection using Stata.

## Results

**Fig. 1:**
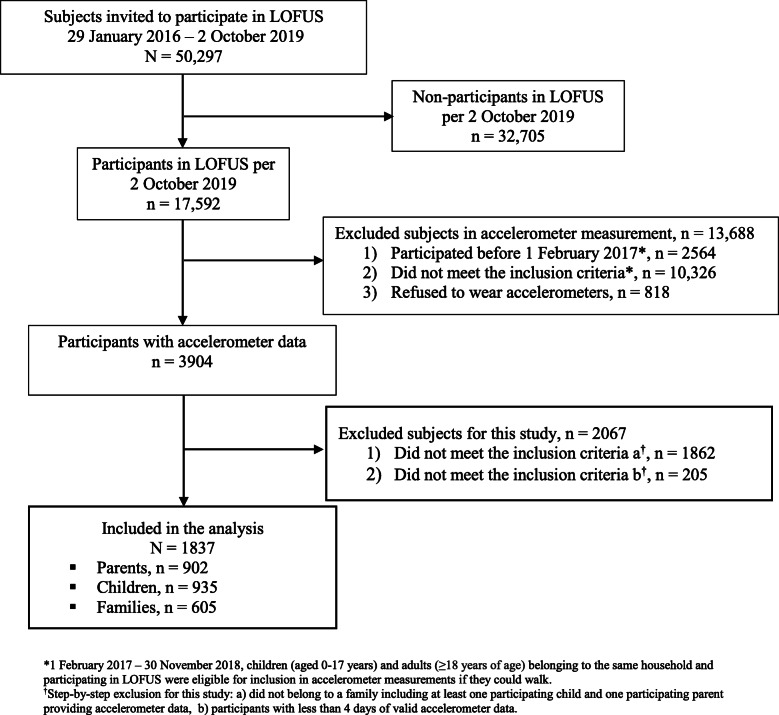
Flow chart for the study on resemblance of physical activity in families with children, Lolland-Falster Health Study (LOFUS)

Figure 1 presents the flow chart of the study. Of the 3904 LOFUS-participants who provided accelerometer data by 2 October 2019, 1837 participants nested within 605 families met the inclusion criteria for the present study. Of these, 902 were parents (female 58.5%; mean age 42.9 ± 7.1 years), and 935 were children (female 55.0%; aged 10 months-22 years; mean age 11 ± 4.5 years). Characteristics of the study participants are displayed in Table 1. The majority of the parents were married or cohabiting (92.0%), only 10.4% had no formal education, and 86.5% were employed, while 5.7% were students and 7.8% were out of work. The number of family members, who were included in the present study, ranged from 2 to 6 members (mean 3 members) (Table 2).

**Table 1:** Characteristics of the sample of parents and children nested in 605 families, *N* = 1837

	n (%)	Mean ± SD
**Parents,** ***n*** **= 902**		
Gender		
Male	374 (41.5)	
Female	528 (58.5)	
Age (years)		42.9 ± 7.1
Body mass index		26.9 ± 5.2
Civil status, *n* = 708		
Married/cohabiting	652 (92.0)	
Divorced/separated/single/widow(er)	56 (8.0)	
Educational level, *n* = 713		
Medium or long higher education	290 (40.7)	
Short higher education or vocational education	349 (48.9)	
One or multiple shorter courses or no formal education	74 (10.4)	
Occupation, *n* = 825		
Employed	710 (86.5)	
Student	49 (5.7)	
Out of work	66 (7.8)	
Physical activity		
LPA (minutes/day)^a^		212.6 ± 56.0
MVPA (minutes/day)^b^		25.5 ± 16.0
VPA (minutes/day)^c^		5.5 ± 6.8
**Children,** ***n*** **= 935**		
Gender		
Boys	420 (45.0)	
Girls	515 (55.0)	
Age (years)		11 ± 4.5
Physical activity		
LPA (minutes/day)^a^		197.5 ± 50.0
MVPA (minutes/day)^b^		56.8 ± 34.0
VPA (minutes/day)^c^		23.7 ± 19.8

^a^*LPA* Light physical activity, ^b^*MVPA* Moderate-to-vigorous physical activity, ^c^*VPA* Vigorous physical activity

**Table 2:** Number of family members per family included in the study

Number of family members	Families, n	Children in the families, n
2	199	199
3	218	315
4	150	297
5	29	85
6	9	35

For parents and children combined, the mean wear time was 21.9 ± 1.2 h per day, while the average accelerometer time included in the processed data was 17 ± 0.8 h per day. The majority of PA was accumulated in LPA for both parents (212.6 ± 56.0 min per day) and children (197.5 ± 50.0 min per day). Parents accumulated on average 25.5 ± 16.0 min per day of MVPA. Almost all this activity was of moderate intensity with only few minutes accumulated in VPA (5.5 ± 6.8 min per day). Children accumulated on average 56.8 ± 34.0 min of MVPA per day. Almost half of this activity was VPA (23.7 ± 19.8) (Table 1).

### Intra-family resemblance in PA

Table 3 shows the clustering in PA within the total family, within parent-child dyads, and within parent-child dyads by the age of the children. For the total family, the ICCs for the various intensities of PA and PA behaviours ranged from 0.06 to 0.34. The intra-family resemblance was stronger in LPA/PA behaviours requiring low energy expenditure (ICCs from 0.21 to 0.34) than in MVPA and VPA (ICCs 0.06 and 0.07, respectively). The ICC for running was lower than for walking (ICC 0.12 versus 0.24, respectively). We found similar results in the age-specific analyses, and the findings from the gender-specific analysis (Table 4) showed a similar pattern independent of gender of parents and children. The ICC for biking increased by the age of the children (ICC 0.03 in age-group 0–6 years versus ICC 0.36 in age-group 12–22 years). It was 0.23 for the total family (Table 3).

**Table 3:** Mixed linear regression analysis of resemblance of physical activity within families, within parent-child dyads, and within parent-child dyads by the age of the child^a^, *N* = 1837

	The total family (*N* = 1837)			Parent-child (dyads *n* = 558)			Age group 0–6 y (dyads *n* = 126)			Age group 7–11 y (dyads *n* = 257)			Age group 12–22 y (dyads *n* = 271)		
	ICC	95% CI	*p* value	ICC	95% CI	*p* value	ICC	95% CI	*p* value	ICC	95% CI	*p* value	ICC	95% CI	*p* value
CPM	0.12	(0.08; 0.19)	< 0.01	0.02	(0.00; 0.62)	0.32	0.08	(0.01; 0.48)	0.39	0.03	–	–	0.06	(0.01; 0.36)	0.01
LPA	0.22	(0.17; 0.28)	< 0.01	0.19	(0.12; 0.29)	< 0.01	0.04	(0.00; 0.82)	0.36	0.17	(0.08; 0.32)	< 0.01	0.17	(0.08; 0.32)	< 0.01
MVPA	0.07	(0.03; 0.14)	< 0.01	< 0.01	–	–	< 0.01	**–**	–	0.06	(0.01; 0.36)	0.23	0.12	(0.05; 0.29)	0.16
VPA	0.06	(0.02; 0.13)	0.01	< 0.01	–	–	< 0.01	**–**	–	0.02	**–**	–	0.07	(0.01; 0.33)	0.02
Sitting	0.21	(0.16; 0.27)	< 0.01	0.13	(0.07; 0.24)	< 0.01	0.10	(0.02; 0.43)	0.01	0.19	(0.10; 0.34)	< 0.01	0.06	(0.01; 0.35)	0.01
Lying	0.22	(0.17; 0.28)	< 0.01	0.23	(0.16; 0.32)	< 0.01	0.30	(0.16; 0.48)	0.01	0.19	(0.10; 0.34)	< 0.01	0.17	(0.09; 0.32)	< 0.01
Sitting + lying	0.34	(0.28; 0.40)	< 0.01	0.33	(0.26; 0.41)	< 0.01	0.31	(0.18; 0.49)	< 0.01	0.33	(0.23; 0.44)	< 0.01	0.25	(0.16; 0.38)	< 0.01
Standing	0.25	(0.19; 0.31)	< 0.01	0.16	(0.09; 0.27)	< 0.01	0.15	(0.04; 0.40)	0.20	0.24	(0.16; 0.34)	< 0.01	0.16	(0.07; 0.31)	0.06
Walking	0.24	(0.19; 0.30)	< 0.01	0.22	(0.14; 0.31)	< 0.01	0.24	(0.11; 0.44)	0.01	0.39	(0.29; 0.49)	< 0.01	0.12	(0.04; 0.30)	< 0.01
Running	0.12	(0.08; 0.18)	< 0.01	0.05	(0.01; 0.24)	0.12	0.15	(0.04; 0.40)	0.23	0.05	(0.00; 0.42)	0.40	0.08	(0.02; 0.30)	0.03
Biking	0.23	(0.18; 0.29)	< 0.01	0.24	(0.17; 0.33)	< 0.01	0.03	–	–	0.25	(0.16; 0.39)	< 0.01	0.36	(0.26; 0.47)	< 0.01

^a^Adjusted for sex, age, parental education, and sex*age

Significant *p*-values ≤0.05

*ICC* Intraclass correlation coefficient, *CI* Confidence interval, *CPM* Counts per minute, *LPA* Light physical activity, *MPA* Moderate physical activity, *MVPA* Moderate-to-vigorous physical activity, *VPA* Vigorous physical activity

**Table 4:** Mixed linear regression analysis of resemblance of physical activity within gender-specific parent-child dyads^a^, *N* = 1837

	Father-daughter (dyads *n* = 211)			Father-son (dyads *n* = 212)			Mother-daughter (dyads *n* = 313)			Mother-son (dyads *n* = 266)		
	ICC	95% CI	*p* value	ICC	95% CI	*p value*	ICC	95% CI	*p* value	ICC	95% CI	*p* value
CPM	0.13	(0.04; 0.33)	0.03	0.03	–	–	0.08	(0.02; 0.28)	0.09	< 0.01	–	–
LPA	0.19	(0.09; 0.35)	< 0.01	0.20	(0.10; 0.37)	< 0.01	0.17	(0.09; 0.31)	< 0.01	0.13	(0.05; 0.30)	0.02
MVPA	0.13	(0.04; 0.33)	0.04	< 0.01	–	–	< 0.01	–	–	< 0.01	–	–
VPA	0.06	(0.00; 0.44)	0.21	< 0.01	–	–	< 0.01	–	–	< 0.01	–	–
Sitting	0.12	(0.04; 0.33)	0.04	0.08	(0.01; 0.36)	0.16	0.19	(0.11; 0.32)	< 0.01	0.11	(0.04; 0.29)	0.04
Lying	0.20	(0.10; 0.36)	< 0.01	0.20	(0.10; 0.36)	0.01	0.22	(0.14; 0.35)	< 0.01	0.29	(0.19; 0.41)	< 0.01
Sitting + lying	0.33	(0.22; 0.46)	< 0.01	0.27	(0.17; 0.42)	< 0.01	0.31	(0.22; 0.42)	< 0.01	0.25	(0.16; 0.38)	< 0.01
Standing	0.16	(0.07; 0.34)	0.01	0.13	(0.04; 0.33)	0.09	0.22	(0.13; 0.34)	< 0.01	0.14	(0.05; 0.30)	0.01
Walking	0.23	(0.12; 0.38)	< 0.01	0.17	(0.07; 0.34)	0.02	0.27	(0.19; 0.39)	< 0.01	0.23	(0.14; 0.36)	< 0.01
Running	0.12	(0.04; 0.33)	0.04	0.09	(0.02; 0.33)	0.16	0.08	(0.02; 0.28)	0.07	0.06	(0.01; 0.34)	0.16
Biking	0.27	(0.16; 0.41)	< 0.01	0.26	(0.15; 0.40)	< 0.01	0.17	(0.08; 0.30)	< 0.01	0.25	(0.16; 0.38)	< 0.01

^a^Adjusted for sex, age, parental education, and sex*age

Significant p-values ≤0.05

*ICC* Intraclass correlation coefficient, *CI* Confidence interval, *CPM* Counts per minute, *LPA* Light physical activity, *MVPA* Moderate-to-vigorous physical activity, *VPA* Vigorous physical activity

## Discussion

In this study, we examined the degree of resemblance in PA within the total family and within pairs of parents and children. We found that parents and children’s time spent sitting/lying, walking, and biking and time spent in LPA had moderate resemblance within families, whereas engagement in non-specific PA with higher intensities showed small or close-to-zero resemblance.

The level of PA of parents and children may differ as a function of the age of the child [35, 36], and some studies have reported that the association between parent and child PA weakens as children grow older [37, 38]. However, our results showing similar intra-family clustering of PA across the age of children correspond to the conclusion of previous reviews [12, 39].

The present study confirms the systematically summarized findings of a recent review, which showed that parent-child resemblance in PA tend to be similar across the gender of parents and children [12]. In this research field, most previous studies have only examined the association between mother and child PA [40], but studies including both mothers and fathers have shown mixed results. Moore et al. [41] found that fathers’ activity level was more strongly associated with the PA level of 4–7 year old children than that of mothers, while Jago and colleagues [42] reported the opposite in a sample of 5–9 year old children. Fisher et al. [43] found no association between parent and child PA regardless of the gender of the child, whereas Abbott and colleagues [44] observed that parental PA was associated with the PA of girls, but not boys in data on pre-school aged children. The inconsistent findings may be explained by methodological weaknesses in these studies such as difference in processing of raw accelerometer data [12]. However, societal and cultural differences in parenthood between study settings may also be part of the explanation. Our finding may reflect that parenthood is more equal between genders in Denmark compared to many other countries. Maternal employment rates are high, and fathers have fewer working hours per week compared to, for example, USA, Australia, and Italy, and Danish parents are therefore more likely to share child-care responsibilities [45]. Furthermore, analyses comparing mornings versus afternoons of weekdays and weekdays versus weekends, respectively may reveal stronger resemblance in parent-child PA in time segments of the week, during which they are likely to be together [46]. More fine-grained studies like this may also provide a deeper understanding of age- and gender-specific differences.

A recent synthesis of data showed a weak positive association between parent and child MVPA [12], which has been the primary outcome in most previous studies. Our results are consistent with this finding, which indicates that the MVPA of family members is under stronger influence by individual [47, 48] and/or extra-family factors rather than factors within the family [49]. However, our findings are not supported by original studies by Fisher et al. [43] including children aged 7–9 years and Heitzler and colleagues [50] including children aged 11–17 years, which found no association between parent and child MVPA. This inconsistency may be due to methodological differences, e.g. use of questionnaires for assessment of parental PA [51] and the lack of a gold standard regarding data reduction of raw accelerometer data [52]. For instance, the varying use of thresholds among studies could add misclassification among the different intensity domains [52].

Our finding of a stronger intra-family resemblance for LPA than for MVPA adds to mixed findings in the prevailing literature. However, a review of studies on the association between parent and child PA found that LPA was rarely used as a PA outcome and only in studies including pre-school aged children. Results of these studies were mixed [12]. Thus, the present study provides new knowledge about the resemblance of LPA in families across the age span of children. Our finding indicates that there are important similarities among family members in time spent in LPA and PA behaviours requiring low energy expenditure (sitting, standing, and lying). This indicates that intra-family factors such as shared environment, co-participation, and family rules [10, 53, 54] influence this kind of non-structured activities [7]. Activities of low intensity may be the easiest to perform together as a family, because they require a similar amount of energy of each family member regardless of age compared to activities such as running, which demands a higher relative energy expenditure of a child than of an adult [55].

The intra-family clustering of biking, which increased by the age of the children, was an interesting finding of the present study, which to the best of our knowledge has not been shown before. Cycling is common in Denmark across age-groups and gender [56, 57] and is widely used for e.g. transportation to school or work and for recreation [57]. Our finding suggests that cycling habits to a moderate extent co-occur among family members, and that in particular older children’s cycling habits resemble those of their parents.

Besides shared social environments and habits, our finding of clustering of PA within families might also be influenced by genetics [11, 58, 59]. However, we included not only nuclear families, but also e.g. blended families without biological ties between all family members. We did not have the opportunity to conduct separate analyses for biologically related parent-child pairs and non-biologically related pairs, and therefore we cannot contribute more specifically to elucidate the effect of genetics in this article.

Our finding that the level of intra-family resemblance ranged from close-to-zero to moderate reflects that other factors apart from the family influence individual PA levels. On the individual level, psychological traits such as temper [60] and self-efficacy [61] may be influential factors, and on the inter-relational level, peers may play an increasingly important role as children mature [38]. Pre-schools [62], schools [63], and workplaces [64], environmental factors in the local community [11, 65], and policy-related factors may all promote or impede PA of individuals [1].

### Strengths and limitations

The present study has several strengths. First, the population-based sampling and the large study sample provided statistical power to uncover potential intra-family resemblance in PA across the full age-range of children and all parent-child gender combinations. However, the relatively small number of 0–6 year old children may be a limitation, especially taking the substantial developmental changes children undergo in early childhood into consideration [66]. Therefore, the results regarding the youngest age-group should be interpreted with caution. Second, this study provides new insight in terms of intensities other than MVPA. Third, the use of a dual-accelerometer system to assess PA in both parents and children allowed device-based reporting of PA over 24 h and accurate classification of time spent in different PA behaviours under free-living conditions [32]. A drawback in the use of accelerometers is that there is no consensus about the method used in the data reduction process, which still makes it difficult to compare results between studies [52].

A possible limitation is that participation in LOFUS and the accelerometer assessment, respectively was voluntary for each family member, and thus, we included incomplete families in our study. This may provide uncertainties when comparing age- and gender-specific sub-groups. We were unable to standardise the size of family (number of parents and children), and instead, we used randomly selected parent-child dyads as analysis units. We do not know why some household−/family members chose not to participate in LOFUS or in accelerometer measurement, respectively, but it could potentially affect the results by limiting generalizability of the findings. An analysis of socio-economic determinants of participation among adults halfway through the LOFUS data collection showed that being middle-aged, female, Danish citizen, and of higher socio-economic status increased participation [67]. Further, the voluntary participation in accelerometer measurement may have increased the selection bias. However, in studies using data from large health surveys, biased participation may not interfere much with the associations between variables [68, 69]. Because the present study was cross-sectional, we cannot assume any causal relationships among the variables. Nevertheless, it is highly probable that the relationship between parent and child PA is bi-directional, so that all family members contribute to the each other’s PA level and PA behaviours.

## Implications for research and public health

More studies of high quality [12] are needed to confirm our results, but also to examine the role of e.g. siblings on children’s PA. In addition, studies on PA in separate time segments of the week and in different cultural settings may provide additional insight. Enriching future studies with data on underlying factors, such as encouragement, support, and modelling for PA may contribute to a more comprehensive picture of the complexity of PA behaviour in the family setting.

Our results indicate that public health initiatives targeting PA habits of families could aim at replacing sedentary activities with activities of light intensity, since these activities seem to have a stronger within-family resemblance than MVPA and VPA. Thus, targeting parental sedentary behaviours as a means to decrease sedentary time and increase LPA in their children might be a possible candidate for intervention. Furthermore, since variation in children’s MVPA and VPA seems not to be explained by the PA of parents, pre-schools and schools may be more fruitful arenas for increasing the PA of children than the home environment, which may also have the potential to level out possible influence of socio-economic factors. Local facilities for sports, play, and active transportation may also be important settings for PA promotion in both adults and children. However, for children, parents may still play an important role through support and motivation for PA.

## Conclusion

This cross-sectorial population study adds to the evidence on the importance of family for PA of family members with a special focus on the parent-child relationship. We found varying degrees of intra-family clustering of PA dependent on the intensity and type of PA, which was similar across the age of children and the gender of parents and children. The strongest similarities were found for LPA and sitting/lying, walking, and biking with moderate resemblance in time spent on these activities between parents and children, whereas engagement in activities with moderate or high intensity showed small or close-to-zero resemblance. More research is required to fully understand factors influencing PA as a complex behaviour in the family setting, but public health interventions targeting the PA habits of families may be fruitful.

## Data Availability

Data used for this study were derived from the Lolland-Falster Health Study (LOFUS). Research groups can apply to the LOFUS steering group for access to use LOFUS data. Each project must adhere to the rules and regulations on research ethics and data protection.

## Abbreviations

CI: Confidence interval
ICC: Interclass correlation coefficient
LOFUS: Lolland-Falster Health Study
LPA: Light physical activity
MPA: Moderate physical activity
MVPA: Moderate-to-vigorous physical activity
NMT: Non-moving temperature
PA: Physical activity
SD: Standard deviation
VPA: Vigorous physical activity
